# Mutational analysis of *PRNP* in Alzheimer’s disease and frontotemporal dementia in China

**DOI:** 10.1038/srep38435

**Published:** 2016-12-02

**Authors:** Weiwei Zhang, Bin Jiao, Tingting Xiao, Chuzheng Pan, Xixi Liu, Lin Zhou, Beisha Tang, Lu Shen

**Affiliations:** 1 Department of Neurology, Xiangya Hospital, Central South University, Changsha, China; 2 Key Laboratory of Hunan Province in Neurodegenerative Disorders, Central South University, Changsha, China; 3 State Key Laboratory of Medical Genetics, Changsha, China

## Abstract

The *prion protein (PRNP*) gene is associated with prion diseases, whereas variants of the *PRNP* gene may also explain some cases of Alzheimer disease (AD) and frontotemporal dementia (FTD) in Caucasian populations. To determine the prevalence of the *PRNP* gene in patients with AD and FTD in China, we screened all exons of the *PRNP* gene in a cohort of 683 cases (606 AD and 77 FTD) in the Chinese Han population and we detected a novel missense mutation p.S17G in a late-onset AD (LOAD) patient. Furthermore, we analyzed the *PRNP* M/V polymorphism at codon 129, which was previously reported as a risk factor. However, there were no significant differences in genotype and allele frequency either in AD (OR = 0.75[0.378–1.49], P = 0.492), or FTD patients (OR = 2.046[0.265–15.783], P = 0.707). To our knowledge, this is the first study to reveal a correlation between the *PRNP* gene and Chinese AD and FTD patients in a large cohort. This study reports a novel p.S17G mutation in a clinically diagnosed LOAD patient, suggesting that the *PRNP* mutation is present in Chinese AD patients, whereas, M129V polymorphism is not a risk factor for AD or FTD in the Chinese Han population.

Alzheimer’s disease (AD) is the most common form of dementia, characterized by progressive episodic memory loss, decline in learning and daily living activities, and finally, comprehensive cognitive domain damage. Over 95% of patients develop the disease after the age of 65 years, named late-onset AD (LOAD), the pathogenesis of which is complex and associated with aging, environmental factors, genetics and other unknown causes^1^. Approximately 1–5% of AD cases are diagnosed before 65 years old, named early-onset AD (EOAD). Genetics is the main contributor to the pathogenesis of EOAD. Family aggregation is more obvious among EOAD patients. Approximately 10% of EOAD patients have a positive family history, and most inherit in an autosomal dominant manner^2^. In general, the heritability of AD is estimated to be up to 79% based on twin and family studies^3^. Three genes involved in amyloidogenic processing have been identified as causative genes of early-onset familial AD (EOFAD): presenilin 1 (*PSEN1*), presenilin 2 (*PSEN2*), and amyloid precursor protein (*APP*) genes^4^. The ε4-allele of the apolipoprotein E gene is a major genetic risk factor for AD^5^,^6^.

Frontotemporal dementia (FTD) is the second most common form of presenile dementia after AD, comprising 10–20% of all forms of dementia^7^. Clinically, FTD exhibits as three forms: behavioral variant FTD (bvFTD), semantic dementia (SD), and progressive non-fluent aphasia (PNFA)^8^. Approximately 40% of FTD patients have a positive family history. A total of 8 genes have been identified as causative genes of both familial and sporadic FTD patients, including microtubule-associated protein tau (*MAPT*)^9^, progranulin (*GRN*)^10^, chromosome 9 open-reading frame 72 (*C9orf72*)^11^, chromatin modifying protein 2B (*CHMP2B*)^12^, TAR DNA-binding protein (*TARDBP*)^13^, valosin-containing protein (*VCP*)^14^, fused in sarcoma (*FUS*)^15^, and coiled-coil-helix-coiled-coil-helix domain containing 10 (*CHCHD10*)^16^.

Rare high-penetrant mutations in causal genes explain only a small fraction of AD and FTD cases, whereas the hereditary factors for most patients, particularly the sporadic form, remain unknown. A hypothesis suggests that a certain portion of patients diagnosed as AD or FTD may have mutations in genes involved in other neurodegenerative brain diseases (NBDs)^17^,^18^.

*Prion protein (PRNP*) is a causal gene of familial Creutzfeldt–Jakob disease (CJD), Gerstmann–Straussler–Scheinker disease (GSS), and fatal familial insomnia (FFI), accounting for 5–10% of inherited forms of prion diseases. Prion diseases share some overlaps with AD and FTD. Clinically, both may exhibit a dementia phenotype during progress of the disease and the symptoms usually progress gradually. They all can be divided into two forms: the sporadic and the familial. The sporadic form is the majority and the familial form is fully penetrant. Pathologically, AD, FTD and CJD are all neurodegenerative diseases, or conformational disorders caused by a common pathogenesis of the excessive accumulation of abnormal, insoluble proteins, including the accumulation of Aβ in AD, tau in FTD and prion protein (PrP^c^) in CJD. Recent studies on the neurotoxic effect of Aβ revealed that PrP^c^ was required for Aβ oligomer-induced neuronal cell death, indicating that PrP^c^ may be involved in the pathogenesis of AD^19^. Genetically, mutations of *PRNP* were found in clinically diagnosed AD and FTD patients, and the homozygosity of codon 129 (M/M alleles) was associated with higher risk for CJD, whereas the heterozygosity of codon 129 (M/V alleles) is a possible risk factor for AD and may modify the age at onset of FTD in some Caucasian populations^20^,^21^.

In our previous study, we screened the *PSEN1, PSEN2,* and *APP* genes in probands of EOFAD families, and the *MAPT, GRN,* and *C9orf72* in FTD patients of the Chinese Han population. We found that the frequency of AD causative genes was 17.1% for *PSEN1*, 5.7% for *APP* and no mutation for *PSEN2*^22^. Mutations in *MAPT, GRN,* and *C9orf72* accounted for 4.3%, 1.4%, and 2.9%, respectively, of FTD cases in the Chinese population^23^. Our previous study revealed that the remaining dementia patients were genetically unexplained and comprised a large group. In this study, we screened the *PRNP* gene in the remaining genetically unexplained AD and FTD patients to elucidate their missing genetics. To our knowledge, this is the first study of the distribution of the *PRNP* gene in a large cohort of Chinese AD and FTD patients.

## Results

The demographic features of 606 AD cases, 77 FTD cases and 523 controls are shown in Table 1. A total of 73 AD patients (12.04%) and 11 FTD patients (13.58%) had a positive family history. No statistically significant differences in sex distribution or age at onset were found between cases and controls. The polymorphic codon 129 of the *PRNP* gene was in Hardy-Weinberg equilibrium in the healthy control (P = 0.76), AD (P = 0.66) and FTD (P = 0.95) population.

### A novel rare variant p.S17G in the *PRNP* gene was identified in a sporadic LOAD patient

**Figure 1:**
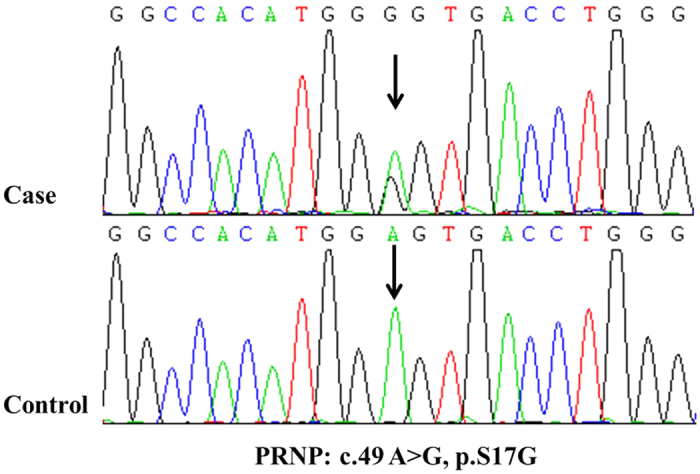
Sequencing chromatograms showing the *PRNP* p.S17G mutation.

**Figure 2:**
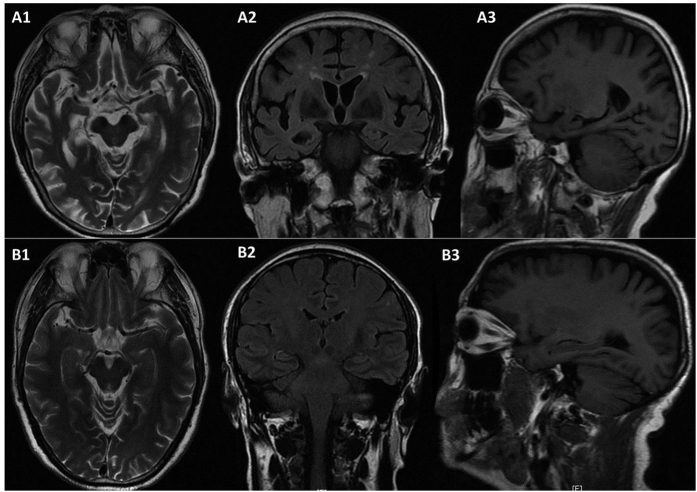
MRI scan of the patient and an age-matched control. (**A1**–**A3**) The cross sectional, coronal, and sagittal MRI of the patient revealed diffuse atrophy of the frontal lobe, temporal lobe and parietal lobe, especially the temporal lobe and the hippocampal. (**B1**–**B3**) The cross sectional, coronal, and sagittal MRI of the age-matched control appeared much normal.

The *PRNP* p.S17G variant was identified in one of 606 AD cases and in none of 534 controls and public databases, including 1000 G, dbSNP, and ExAC (Fig. 1). The carrier had the M129M genotype and the ApoE genotype was ε2/3. The patient was a 70-year-old female. She first developed episodic memory loss 5 years ago, particularly short-term memory. She easily forgot tasks and words she had spoken. Over the next 2 years, her symptoms worsened with a gradual loss of calculation abilities, orientation in time and space, and perceptivity. She had difficulty taking care of herself as well as dealing with daily tasks. She also presented with personality changes characterized by irritability, depression and apathy, and barely talked to others. Neurological examination revealed no myoclonic jerks, seizures, extrapyramidal or upper motor neuron signs apart from the memory impairment and cognitive deficits. Her Mini-Mental State Examination (MMSE) and Montreal Cognitive Assessment (MoCA) scores were 2/30 and 0/30, respectively, the Activity of Daily Living Scale (ADL) score was 54, and the Clinical Dementia Rating (CDR) score was 3. The MRI revealed diffuse cortical atrophy, enlargement of the cerebral ventricle and cistern of the whole brain, especially in the frontotemporal lobe, and hippocampus (Fig. 2). She was diagnosed with probable AD according to the NINCDS-ADRDA criteria. No family members suffered from dementia, and the available DNA sequencing of her family members revealed no *PRNP* p.S17G mutation.

### The M129V genotype may not be a risk factor for AD and FTD in the Chinese Han population

To assess the correlation between the *PRNP* M/V polymorphism at codon 129 and the susceptibility to AD in mainland China, we examined the genotype and allele frequencies of this polymorphism in 606 Chinese Han AD patients and in 534 healthy controls. The genotype distribution of codon 129 polymorphisms in the AD group was 96.5% MM and 3.5% MV, 98.7% MM and 1.3% MV in the FTD, and 97.4% MM and 2.6% VV among controls. No significant difference between AD patients (including EOAD and LOAD) and the controls were found for genotype or allele frequency of the *PRNP* 129 polymorphism (OR = 0.714[0.285–1.792], P = 0.316) (Table 2). We also investigated the genotype and allele frequencies of *PRNP* 129 in 77 Chinese FTD patients to determine whether this polymorphism correlated with FTD. There were no significant differences in the genotype and allele frequencies between FTD patients and controls (OR = 1.949[0.231–16.444], P = 0.461) (Table 2). This result suggests that the *PRNP* M/V polymorphism at codon 129 may not increase the susceptibility to AD or FTD.

## Discussion

Located on chromosome 20p13, the *PRNP* gene encodes the prion protein containing 253 amino acids. Thus far, more than 50 mutations in *PRNP* have been reported to cause autosomal dominant inherited CJD, and a large number of polymorphisms in *PRNP* (including codon 129, 219, 178, 200, and 232, etc.) have been identified as susceptible factors in sporadic CJD (sCJD) cases. For example, the M/M genotype of codon 129 is associated with a higher portion of sCJD, early-onset and a shorter incubation time of kuru. Due to the overlap of clinical phenotypes and pathological characteristics between CJD and other neurodegenerative disorders, a series of studies supported the hypothesis that mutations and polymorphisms of *PRNP* also accounted for other neurodegenerative diseases, including AD, FTD, Parkinson’s disease (PD) and primary progressive aphasia^24^,^25^,^26^. For example, the nonsense mutation p.Q160* and p.Y145* in *PRNP* were reported in clinically diagnosed AD cases^27^,^28^. A missense mutation p.P39L was identified as causative in an FTD family^29^, and the M/V polymorphism at codon 129 was demonstrated to confer a higher risk to AD in several European studies, but yielded controversial results in populations in Japan and Korea^30^,^31^. Given the clinical heterogeneity of *PRNP*, we propose that the *PRNP* gene may also account for Chinese AD and FTD patients. In this study, we screened 606 AD patients, 77 FTD patients and 534 age-matched controls to determine whether the *PRNP* gene is causative in the Chinese AD and FTD population. We also examined the correlation between the *PRNP* M/V polymorphism at codon 129 and the susceptibility of AD and FTD in China.

As a result, we identified a novel missense mutation p.S17G of the *PRNP* gene in a LOAD patient. To our knowledge, this is the first reported in the Chinese Han AD population. The mutation was absent in other family members of the carrier and 534 cognitively normal, age-matched controls, suggesting that the p.S17G maybe a de novo, likely pathogenic mutation in this patient. The carrier presented this variation in a heterozygosis status. She started episodic memory loss at the age of 65, and gradually exhibited depression, calculation, orientation and perceptivity disabilities. Neuropsychological assessments were consistent with her clinical manifestations. Cranial MRI screening revealed diffuse cortical atrophy, most seriously in the frontotemporal lobe and hippocampus. Moreover, the clinical course in this patient was not rapidly progressive as the duration of the disease was over 5 years. Unfortunately, we didn’t receive permission from her relative to conduct a brain biopsy; however, based on the clinical course and MRI findings, the patient seems more likely to have AD than CJD. Previous reports of *PRNP* mutations in AD and other dementia phenotype are summarized in Table 3. Thus far, a total of 8 point mutations of the *PRNP* gene have been reported to be associated with the clinical diagnosis of AD, FTD, DLB, and AD/FTD phenotype of GSS and CJD. The reason for the phenotypic heterogeneity of *PRNP* mutant remains unclear; therefore, we propose that AD, FTD and other neurodegenerative dementia maybe a low penetrance phenotype of *PRNP* mutation. The clinically diagnosed AD patients with a *PRNP* mutation appear to share some common features, such as initial symptom of short-memory loss, followed by depression, spatial and temporal orientation, absence of myoclonus and extrapyramidal signs, prolonged clinical courses and a negative family history in a few patients. The phenotype of our patient is consistent with previous reports. The Ser-to-Gly substitution is located in the signal peptide near the N-terminal of the human prion protein and little is known about the exact function of the (1–22) peptide. Studies on the N-terminal (1–28) part of the mouse prion protein revealed that it is a cell penetrating peptide, capable of transporting large hydrophilic cargoes through a cell membrane with a strong tendency for aggregation and beta-structure formation^32^. It will thus be of considerable interest to determine whether this processing peptide influences the transformation and aggregation of the prion protein.

We further examined the M/V polymorphism at codon 129 in our cohort, and failed to find an association in the AD or FTD cohort, even when the AD cohort was divided into EOAD and LOAD subgroups. The frequency of the genotypes of our cohort was MM 96.5% and MV 3.5% in AD, MM 98.7% and MV 1.3% in FTD and 97.4% MM and 2.6% VV among controls. The VV genotype was absent in our cohort. Our results are consistent with the previous reports in Asian and part of Caucasian populations^30^. It is possible that the *PRNP* 129 polymorphism does not affect the risk of AD or FTD in Asians because the V allele is thoroughly rare in both cases and healthy controls. Another possibility is that a false negative result was obtained due to the limited sample size, particularly the FTD cases. However, our study is fundamental for identifying the mutations and polymorphisms of the *PRNP* gene in China. These data will help advance our understanding of *PRNP* gene in the Chinese and East Asian populations.

In conclusion, we found a novel p.S17G mutation in a clinically diagnosed LOAD patient, suggesting that the *PRNP* mutation is present in Chinese AD patients. Further functional studies on the *PRNP* p.S17G and replication in other cohorts are necessary to confirm the pathogenicity of this *PRNP* mutation. Genetic screening of the *PRNP* gene is warranted in neurodegenerative dementia, particularly in suspected AD cases. The V allele of codon 129 is rare in our cohort and the M/V polymorphism at codon 129 was not significantly associated with the incidence of AD and FTD in China.

## Method

### Subjects

This study included 606 AD cases (including 533 sporadic AD and 73 probands from FAD families), 77 FTD patients (including 66 sporadic FTD and 11 probands from FTD families), and 523 sex, age-matched normal controls. All subjects were enrolled from the outpatient neurology clinics of Xiangya Hospital and Jiangsu Geriatric Hospital. The AD cohort met the NINCDS-ADRDA criteria for probable AD, and the FTD cohort met the consensus criteria for bvFTD, SD or PNFA. All patients underwent standard neuropsychological assessments including MMSE, MoCA, CDR, ADL, verbal fluency test, voice fluency test, and Alzheimer’s Disease Assessment Scale-cognitive subscale (ADAS-Cog). The study was approved by the Ethics Committee of Xiangya Hospital, Central South University in China (equivalent to an Institutional Review Board) and performed in accordance with the approved guidelines and regulations. Written informed consent was obtained from each subject. For all patients, careful clinical, neurological examination and blood tests for vitamin status, thyroid function, HIV and Treponema pallidum infection were conducted to avoid the possibility of reversible dementia.

### DNA isolation and genotyping

Genomic DNA was extracted from peripheral blood leukocytes using a QIAGEN kit following the supplier’s instructions. All DNA samples were normalized to 50–100 ng/μl. Polymerase chain reaction (PCR) was performed on the exonic regions of PRNP (NM_001080123), as well as their corresponding flanking intronic sequences. All PCR products were sequenced using identical forward and reverse primers with BigDye terminator v3.1 sequencing chemistry on an ABI 3730xl DNA analyzer (Applied Biosystems). DNA sequences were analyzed using the Sequencher software. All related primer information and protocols are presented in the Supplementary materials.

Patients with mutations of *APP, PSEN1, PSEN2, MAPT, GRN* and *C9orf72* were excluded from the study. The prevalence of all novel mutations and polymorphisms at codons were further tested in 534 healthy individuals of matched geographical ancestry and age to provide further evidence of pathogenicity. The control group comprised volunteers from communities and the health examination center. All control individuals were older than 65 years and had scores indicative of no cognitive impairment (>26/30) on the MMSE.

### Statistics analysis

According to the polymorphism at codon 129, patients and controls were classified into three groups as follows: MM, MV and VV genotype. Differences in the distribution of polymorphisms at codon 129 between the patients and controls were tested using Pearson χ2 test or Fisher’s exact test when the sample size was small, and significance was set at P = 0.05. Descriptive statistics were expressed as the mean ± the standard deviation. The associations between polymorphisms at codon 129 and disease risk were determined in logistic regression models adjusted for the age at onset and gender. Statistical analysis was performed using SPSS Statistics software (version 21.0).

## Additional Information

**How to cite this article**: Zhang, W. *et al*. Mutational analysis of *PRNP* in Alzheimer’s disease and frontotemporal dementia in China. *Sci. Rep.* **6**, 38435; doi: 10.1038/srep38435 (2016).

**Publisher's note:** Springer Nature remains neutral with regard to jurisdictional claims in published maps and institutional affiliations.

## Supplementary Material

Supplementary Information

## Figures and Tables

**Table 1:** Demographic data of AD patients and control groups.

Variable	AD	AD		FTD	Control
		EOAD	LOAD		
Cases, n	606	170	436	77	534
Sex, male(%)	275(45.3%)	77(45.3%)	198(45.4%)	36(46.8%)	262(49.1%)
Age	70.72 ± 10.83	55.48 ± 8.97	75.38 ± 5.74	58.43 ± 15.25	68.3 ± 11.6
Age at onset	67.16 ± 10.68	51.76 ± 8.27	71.84 ± 5.57	55.57 ± 14.52	—
MMSE score	18.05 ± 7.96	16.38 ± 7.195	18.85 ± 8.239	16.29 ± 9.53	28.6 ± 1.7

**Table 2:** Genotypic and allelic distribution of *PRNP* codon 129 in AD, FTD patients and controls.

	Genotype			Allele	
	MM	MV	VV	M	V
Control	520	14	0	1054	14
AD	585	21	0	1191	21
FTD	76	1	0	153	1
FTD vs Control					
OR	2.046			2.032	
95%CI	0.265–15.783			0.265–15.564	
P value	0.707			0.709	
AD vs Control					
OR	0.75			0.753	
95%CI	0.378–1.49			0.381–1.489	
P value	0.492			0.496	
EOAD vs Control					
OR	0.888			0.789	
95%CI	0.315–2.504			0.318–2.489	
P value	0.789			0.79	
LOAD vs Control					
OR	0.707			0.711	
95%CI	0.341–1.465			0.345–1.464	
P value	0.358			0.362	

**Table 3:** Mutations in *PRNP* reported in dementia population.

Mutation	AAO	Clinical diagnosis	Clinical symptoms	Disease duration	Family history	CT/MRI	Brain autopsy	Reference
p.I215V	75	AD	Memory loss, spatial and temporal orientation, myoclonic episode	4.5 years	Yes	Brain atrophy within the temporal region	abundant senile plaques and neurofibrillary tangles	33
p.Q160*	38	AD	Memory loss, impulsive behavior, temporary diarrhoea	N	Yes	PET: left frontal hypometabolism	N	27
p.Q160*	39	AD	Memory loss, depression,	8 years	Yes	unremarkable	severe NFTs and neuritic plaque-like pathologic, extensive PrP^c^	34
p.Y145*	38	AD	Memory loss, disorientation	Over 12 years	No	Severe brain atrophy with dilation of lateral ventricle	Amyloid deposition in parenchymal and leptomeningeal blood vessel, NFTs in cerebral gray matter	28
p.D178N	50	AD	memory loss, depression, speech and language difficulties, dyspraxia	N	Yes	frontoparietal cortical atrophy	N	35
p.P39L	66	FTD	Apathy, short-memory deficit, postural instability	N	Yes	Bilateral frontal lobe atrophy, confluent lesions in the white matter	N	29
p.P39L	60	FTD	difficulties in word recruitment and writing, short-term memory, attention disabled, transient topographic disorientation, behavioral disturbances and executive dysfunctions	N	No	diffuse cortical atrophy prominent in mesial frontal, temporal, and posterior parietal regions, mainly in the left side	N	36
p.P39L	75	FTD	apathy, reduction in speech, emotional flatness, anhedonia, mental rigidity and perseverative and routine behaviors	3 years	No	cortical atrophy in mesial frontal, temporal, and posterior parietal regions in the left side	N	36
p.M232R	55	DLB	Moderate dementia, ideational apraxia, masked face, tilted gait, mild dysarthria, and rigidity of extremities	7 years	No	mild cortical atrophy	Lewy bodies in the substantia nigra and cerebral cortices, spongiform degeneration, kuru plaques, and abnormal prion aggregates were abscent	37
p.P102L	46	FTD phenotype of GSS	Emotional flattening, difficulty in execution, short-memory loss visual hallucinations, delusions, irritability and aggressive behaviour	More than 6 years	Yes	severe diffuse brain atrophy associated with slightly hyperintense signal in the frontal lobe cortex in FLAIR images	N	38
p.D202G	55	“other disease” regarding CJD	Forgetfulness, mild gait disturbances, difficulties in writing, and hypersomnia	15 years	Yes	cerebellar atrophy	N	39
