# Supplementary material for: Mutational analysis of PRNP in Alzheimer’s disease and frontotemporal dementia in China
Source: Sci Rep. 2016 Dec 2;6:38435. doi: 10.1038/srep38435 (PMC5133586; doi:10.1038/srep38435)
Supplement: Supplementary Information [file srep38435-s1.pdf]

Mutational analysis of *PRNP* in Alzheimer's disease and frontotemporal dementia in China

Weiwei Zhang[1], Bin Jiao[1], Tingting Xiao[1], Chuzheng Pan[1], Xixi Liu[1], Lin Zhou[1, 2], Beisha Tang[1,2,3], Lu Shen[1,2,3,*]

[1] Department of Neurology, Xiangya Hospital, Central South University, Changsha, China

[2] Key Laboratory of Hunan Province in Neurodegenerative Disorders, Central South University, Changsha, China

[3] State Key Laboratory of Medical Genetics, Changsha, China

Correspondence: Dr. Lu Shen

Professor of Department of Neurology

Xiangya Hospital Central South University

87# Xiangya Rd, Changsha 410008

P.R. China

Phone: +86-731-84327623

Fax: +86-731-84327332

Email: shenlu2505@126.com

PRNP primer information and PCR conduction

Briefly, Genomic DNA was PCR amplified using a 10μl reaction including 1μl of 50ng/μl of DNA samples, 0.4μl of dNTP mix (Takara Bio, Inc), 0.4μl of 100ng/μl of forward and reverse primer, 5μl 2×GC BufferⅡ(Takara Bio, Inc), 2.7μl ddH2O, 0.1μl of TaKaRa LA Taq DNA polymerase (Takara Bio, Inc). (forward, reverse primers and reaction conditions are listed in Table 1)

Table 1: The primers and conditions for polymerase chain reaction primers conditions

	primers	conditions
Exon 1	F ACTGAGCAGCTGATACCATTG<br>R TCCGGGACAAAGAGAGAAGA	95 ℃ 5min— (94 ℃ 30s— 62.0 ℃ 30s— 72℃30s) 10cycles (-0.5℃per cycle) — 95℃ 5min – (94℃ 30s—57.0℃30s—72℃30s) -25cycles-72℃ 10min—4℃
